# Deep Learning Models for Predicting Severe Progression in COVID-19-Infected Patients: Retrospective Study

**DOI:** 10.2196/24973

**Published:** 2021-01-28

**Authors:** Thao Thi Ho, Jongmin Park, Taewoo Kim, Byunggeon Park, Jaehee Lee, Jin Young Kim, Ki Beom Kim, Sooyoung Choi, Young Hwan Kim, Jae-Kwang Lim, Sanghun Choi

**Affiliations:** 1 School of Mechanical Engineering Kyungpook National University Daegu Republic of Korea; 2 Department of Radiology School of Medicine Kyungpook National University Daegu Republic of Korea; 3 Department of Internal Medicine School of Medicine Kyungpook National University Daegu Republic of Korea; 4 Department of Radiology Keimyung University Dongsan Hospital Daegu Republic of Korea; 5 Department of Radiology Daegu Fatima Hospital Daegu Republic of Korea; 6 Department of Radiology Yeungnam University Medical Center Daegu Republic of Korea; 7 Department of Radiology School of Medicine Daegu Catholic University Daegu Republic of Korea

**Keywords:** COVID-19, deep learning, artificial neural network, convolutional neural network, lung CT

## Abstract

**Background:**

Many COVID-19 patients rapidly progress to respiratory failure with a broad range of severities. Identification of high-risk cases is critical for early intervention.

**Objective:**

The aim of this study is to develop deep learning models that can rapidly identify high-risk COVID-19 patients based on computed tomography (CT) images and clinical data.

**Methods:**

We analyzed 297 COVID-19 patients from five hospitals in Daegu, South Korea. A mixed artificial convolutional neural network (ACNN) model, combining an artificial neural network for clinical data and a convolutional neural network for 3D CT imaging data, was developed to classify these cases as either high risk of severe progression (ie, event) or low risk (ie, event-free).

**Results:**

Using the mixed ACNN model, we were able to obtain high classification performance using novel coronavirus pneumonia lesion images (ie, 93.9% accuracy, 80.8% sensitivity, 96.9% specificity, and 0.916 area under the curve [AUC] score) and lung segmentation images (ie, 94.3% accuracy, 74.7% sensitivity, 95.9% specificity, and 0.928 AUC score) for event versus event-free groups.

**Conclusions:**

Our study successfully differentiated high-risk cases among COVID-19 patients using imaging and clinical features. The developed model can be used as a predictive tool for interventions in aggressive therapies.

## Introduction

In December 2019, SARS-CoV-2, also called COVID-19, was first detected in Wuhan, China [1]. Since then, the COVID-19 pandemic has rapidly propagated across the world via airborne person-to-person transmission [2,3]. Some patients with COVID-19 progressed to novel coronavirus pneumonia (NCP), which can lead to severe acute respiratory failure, multiple organ failure, and, in some cases, death [4]. A recent study reported that more than 60% of patients who progressed to a severe stage of NCP died [4,5]. Therefore, it is critical to identify high-risk patients among those with advanced COVID-19 to deliver early intensive care.

COVID-19 is diagnosed using viral nucleic acid detection employed by reverse transcription–polymerase chain reaction (RT-PCR) [6]. Although this approach is considered the most effective, it is both time-consuming and has a high rate of false negatives [7]. As an alternative, computed tomography (CT) can be utilized for the initial screening of NCP [8]. CT imaging exhibits the advantage of faster processing time as compared with the molecular diagnostic test. CT scans can also provide detailed structural information, such as the extent of lung involvement and quantitative analysis of NCP lesions associated with prognostic value in patients with COVID-19 [9]. Furthermore, the Fleischer Society has highlighted CT imaging as being crucial in the management of the disease [10]. CT imaging can also be easily performed in a facility-equipped hospital and can assist in the triage assessment of COVID-19 patients by identifying those with severe cases.

Artificial intelligence (AI) methods, particularly deep learning (DL), have shown promising results for lung disease analysis using CT scans. Recent advances via machine learning in the prognosis of COVID-19 patients include estimating the mortality risk in patients with suspected or confirmed COVID-19, predicting progression to a severe or critical state, and predicting the duration of hospital stay [11-15]. Predicting factors included age; features derived from the CT machine; lactate dehydrogenase; sex; C-reactive protein (CRP); comorbidity, including hypertension, diabetes mellitus, cardiovascular disease, and respiratory disease; and lymphocyte count. The advantages and disadvantages of these studies have been described in a recent study by Wynants et al [16]. However, the way of utilizing these models, including data acquisition, was not clearly described and lacked generalization to diverse populations. Some models consider only clinical indicators, demographics, and laboratory tests [17], whereas others only consider CT images [18]. In addition, the timing of the follow-up varies between studies; therefore, the accuracy of the models was not consistent and ranged from 90% to 98% among studies. Featured in these papers was Kang et al [18], who developed an AI system that can diagnose NCP. Furthermore, this system is able to differentiate NCP from common pneumonia and other normal controls using a large CT database of 3777 patients. They used existing networks—3D ResNet (residual neural network)-18, U-Net, DRUNET (dilated-residual U-Net), FCN (fully convolutional network), SegNet (segmentation network), and DeepLabv3—to build two lung-lesion segmentation models and then provide a diagnosis prediction. This system has been tested and has been successfully able to provide diagnoses at several hospitals in China. In addition, a recent study [17] analyzed the electronic health records of patients confirmed to have COVID-19 at a single center in the Mount Sinai Health System in New York City to predict critical events and mortality with a boosted decision tree–based machine learning model. However, their proposed method was based only on the data extracted within 36 hours of patients’ hospitalization, failing to consider clinical parameters during the hospital stay. Furthermore, some patient test parameters are missing from their data set, affecting the final evaluation results. In view of the above problems, we propose a DL algorithm combining an artificial neural network (ANN) and a convolutional neural network (CNN) to build a risk prediction model for all COVID-19 patients. Predicting a personalized prognosis is important for detecting high-risk patients who are more likely to become critical and would require intensive care. In addition, it is crucial to accelerate the development of AI techniques to predict clinical prognosis, particularly during a crisis period caused by the current pandemic.

We hypothesize that a mixed model consisting of both ANN using clinical parameters and 3D CNN using CT imaging—an artificial convolutional neural network (ACNN) model—can help classify patients into event and event-free COVID-19 groups. The events include high-flow nasal cannula, mechanical ventilator care, septic shock, acute kidney injury, continuous renal replacement therapy, extracorporeal membrane oxygenation, intensive care unit admission, or death. The 3D ACNN with CT images can potentially identify the abnormalities of lung parenchyma and clinically predict relevant outcomes in COVID-19 patients. DL models can assist radiologists, physicians, and clinicians in performing a quick diagnosis that can help in decision making and resource allocation, which is particularly important when the health system is overloaded.

## Methods

The institutional review boards of all participating hospitals approved this retrospective study, and the requirement for patient consent was waived.

### Study Population and Image Acquisition

We retrospectively reviewed 330 chest CT scans of COVID-19 patients that were obtained in five hospitals in Daegu, South Korea, from January 31 to April 10, 2020. All patients were confirmed based on RT-PCR tests for SARS-CoV-2 from nasal-pharyngeal swabs. All chest CT scans were performed within 3 days of the COVID-19 diagnosis. A total of 33 patients were excluded from our study owing to the following causes: (1) poor image quality (n=9), (2) insufficient medical records (n=10), (3) no pneumonic infiltration on CT scans (n=8), or (4) failure of image segmentation with both AVIEW (Coreline Soft, Co) and 3D Slicer Chest Imaging Platform (CIP) (Brigham and Women's Hospital), possibly due to the lack of number of slices (n=6). A total of 297 patients were included in our AI analysis. All of the chest CT scans were obtained in the supine position at full inspiration with or without contrast media and performed using one of the following various multidetector CT scanners: SOMATOM Sensation 64, SOMATOM Definition AS/AS+, SOMATOM Definition Flash, or SOMATOM Perspective (Siemens Healthineers); Optima CT660, LightSpeed 16, or Revolution EVO (GE Healthcare); or Aquilion PRIME (Toshiba Medical Systems). The scanning parameters were as follows: a tube voltage of 100-140 kVp, a tube current of 32-192 mAs with a volume CT dose index of 3.97-13.77 mGy, a slice thickness of 1.0-3.0 mm, a detector collimation of 128 × 0.6 mm or 64 × 0.6 mm, and a beam pitch of 1.0-1.2. Axial images were reconstructed with a standard or sharp reconstruction kernel.

### Demographic, Clinical, and Laboratory Data

We analyzed the clinical and laboratory data of each patient at the time of admission from medical records. This included age; sex; smoking history; clinical symptoms; underlying disease, including hypertension, diabetes mellitus, chronic obstructive pulmonary disease, chronic kidney disease, and coronary artery calcification; systolic blood pressure; white blood cell count (WBC); CRP level; respiratory rate; heart rate; and oxygen saturation. To identify high-risk cases among COVID-19 patients, the endpoint was the occurrence of events subsequent to admission.

### ANN Model With Demographic, Clinical, and Laboratory Data

**Figure 1:**
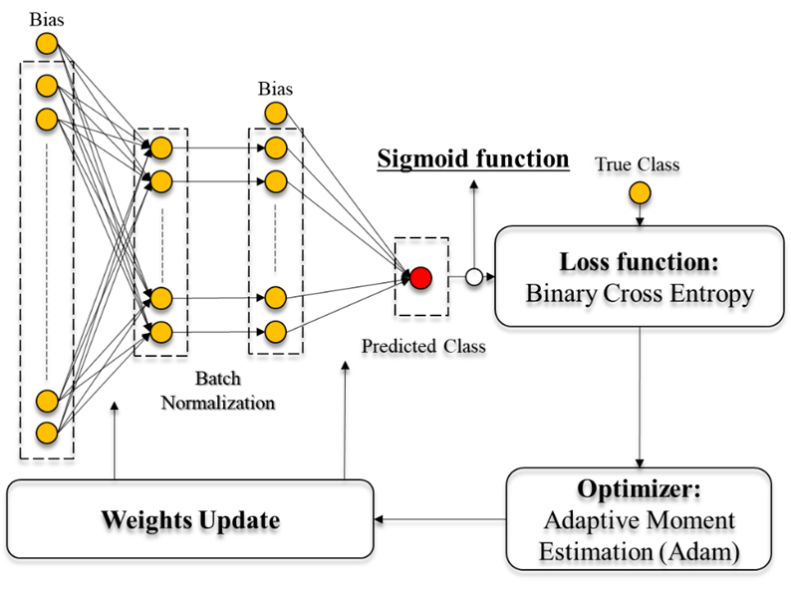
Architecture of a text-based artificial neural network (ANN).

With only clinical and laboratory data, we constructed an ANN model to predict whether input subjects were event or event-free patients. The ANN models comprise nodes, layers, activation functions, optimizers, and loss functions. Nodes between layers are connected by edges along with individual weights (see Figure 1). After the end of one iteration (ie, 1 epoch), with real and predicted classes obtained from the ANN model, a loss function was computed and weights were updated in each layer through the optimizer to minimize the loss. The binary cross-entropy and adaptive moment estimation (Adam) optimizer [19] were used for the loss function and optimizer, respectively. For improving the classification performance, we further added *L*_2_ regularization on the loss function. To prevent a gradient vanishing issue, we used a rectified linear unit (ReLU) function [20] from layer to layer and a sigmoid function at the last layer as activation function. We assessed the number of layers from one to six for the ANN model to determine the optimal number of layers. For an objective comparison, we evaluated all of the ANN models using the same training and testing data. In the end, we obtained an optimized number of layers to achieve the best classification performance compared with the number of other layers.

### CT Image Processing

**Figure 2:**
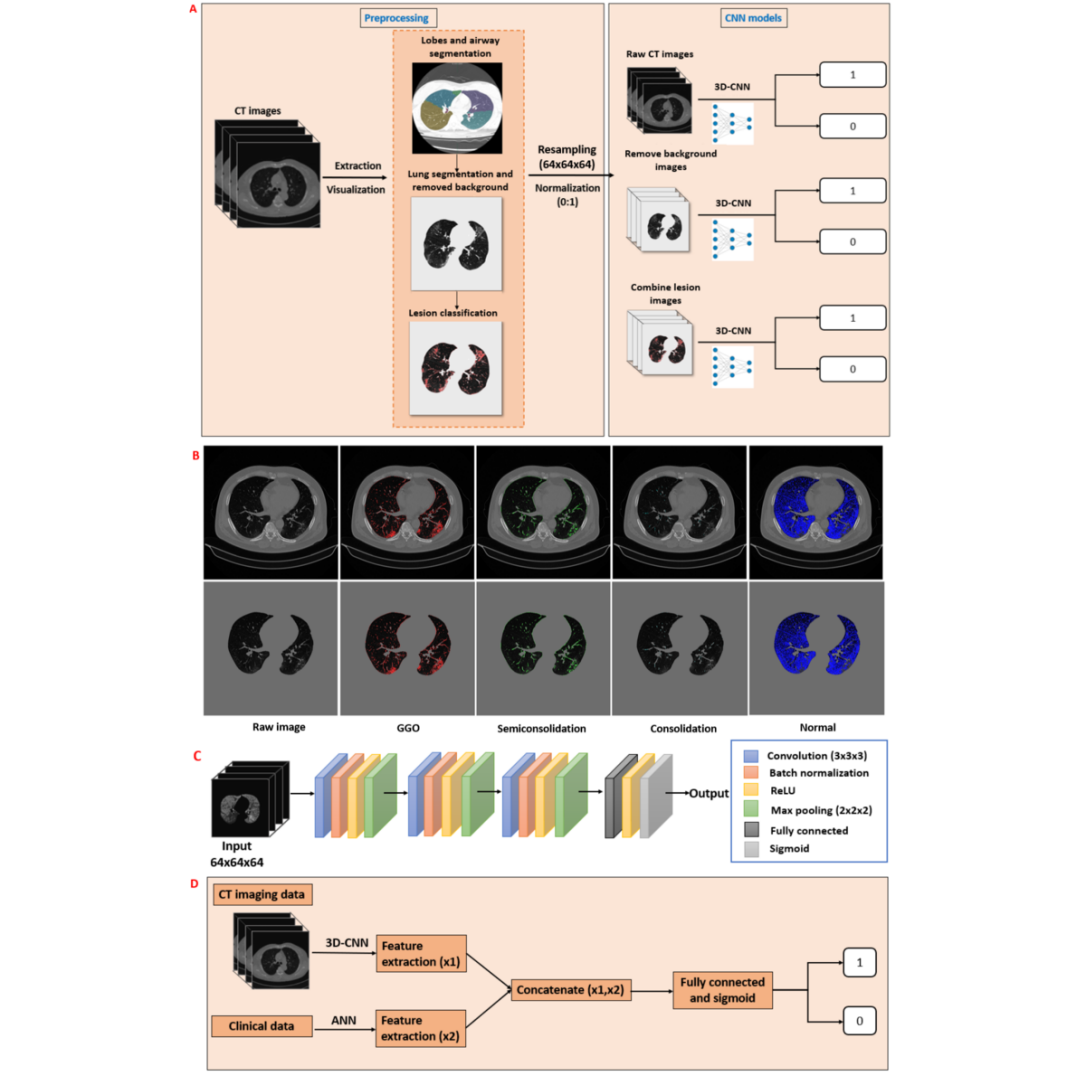
Main experimental products of (A) convolutional neural network (CNN) models, (B) lung lesion segmentation parts of a severe subject, (C) the architecture of our CNN model, and (D) illustration of the artificial convolutional neural network (ACNN) model, a mix of the artificial neural network (ANN) and CNN models. CT: computed tomography; GGO: ground-glass opacity; ReLU: rectified linear unit.

Image segmentation for lungs was performed using the lobes and airway segmentation modules for 288 subjects with AVIEW and for 9 subjects with 3D Slicer CIP [21]. This step separated the voxels corresponding to the lung parenchyma and airway from the voxels corresponding to the surrounding anatomy (ie, mediastinum, thoracic cage, muscle, and space outside the body) of the original CT images (see Figure 2, A). The image obtained through the segmentation process was defined as the lung segmentation image. Next, NCP lesions were identified for detecting abnormal regions using CT Hounsfield unit (HU) thresholds: ground-glass opacity (GGO), consolidation, semiconsolidation, and normal lung. Each component in NCP lesion images is colored using a specific value: normal lung is 8 (color-coded blue; −950 to −701 HU) [22], GGO is 32 (color-coded red; −700 to −501 HU), semiconsolidation is 64 (color-coded green; −500 to −201 HU) [9], and consolidation cluster is 64 (color-coded cyan; −200 to 60 HU); in addition, 8 is set for an unclassified voxel. Figure 2, B visualizes the exemplary distributions of patients experiencing severe-stage COVID-19. These spatial distributions of lesion components were trained for comparison with the raw CT and lung segmentation CT images.

### 3D CNN Models With CT Imaging Data

All of the 297 images were resampled to 64 × 64 × 64 voxels using a linear interpolation method, and the HU of each pixel was normalized to the range between 0 and 1. Figure 2, C illustrates the architecture of our 3D CNN network, which comprised nine layers: three convolutional layers, three batch normalization layers, and three max-pooling layers. After each convolutional layer of a 3 × 3 × 3–kernel size, the feature maps were down-sampled by a max-pooling layer with a 2 × 2 × 2–voxel window. ReLU was used as an activation function to maintain positive input values and change negative input values to zeros in each convolutional layer. The number of filters was determined as 32, 64, and 128, based on our experience. The sigmoid function was then used to distinguish between event and event-free COVID-19 patients using the last fully connected layer. Three typical successful CNN models (ie, ResNet50 [23], InceptionV3 [24], and DenseNet121 [25]) were respectively implemented herein. We have developed these typical 2D models into a 3D domain and trained them to use the same input data set. These models used multiple convolutional blocks with residual connections to continuously extract local and global contextual features. The neural networks were trained using binary cross-entropy between the predicted and true diagnoses as the loss function. The Adam optimization algorithm and the proposed default settings (ie, learning rate=0.001) of the parameters were employed to find the weights of the CNN model [26,27]. The proposed CNN model was also trained for 3000 iterations with a batch size of 16 samples. Moreover, we implemented these typical models into a 2D domain for comparison by extracting one slice per subject at the location representing 50% of the total slices with the input size of 128 × 128 pixels.

### A Mix of CNN and ANN Models: 3D ACNN

We applied the fully connected layer to the last layers of the previously described CNN model to derive a 256-dimensional feature vector to represent a CT image. A total of 19 clinical features of the same patient were concatenated with this feature vector. A new model (ie, ACNN) takes this combined feature vector as the input to predict the patient’s COVID-19 status (see Figure 2, D). A total of 19 clinical features of the same subject were concatenated with a 64-dimensional feature vector of the CT image. The classification conclusions of CNN models still lack transparency and cannot straightforwardly provide reasoning and explanations as do human experts in diagnosis [26]. We used the gradient-weighted class activation mapping (Grad-CAM) [28] approach for visualizing the CNN learning process. This method creates a 2D spatial heatmap as a visual explanation that indicates where the CNN has focused to make its predictions of images, which can track the spatial attention of the CNN when predicting COVID-19 status.

### Validation of AI Models and Statistical Analysis

We used 5-fold cross-validation to evaluate the performance of the ANN, CNN, and ACNN models. We implemented our models using a system on the Intel Xeon Processor E5-2640 v4, 2.40 GHz, with the NVIDIA GeForce RTX 2080 Ti graphics card. We applied a cost-sensitive neural network [29] method to handle our imbalanced dataset (ie, small number of event subjects) using class weighting. The measures of accuracy, precision, sensitivity, specificity, F1 score, confusion matrix, receiver operating characteristic (ROC) curve, and area under the curve (AUC) score were calculated using the true positive (TP), true negative (TN), false negative (FN), and false positive (FP) results [30]. From the confusion matrix, we calculated five values for accuracy, precision, sensitivity, specificity, and F1 score as follows:

*Accuracy* = (*TP* + *TN*) / (*TP* + *TN* + *FP* + *FN*) **(1)**

*Precision* = *TP* / (*TP* + *FP*) **(2)**

*Sensitivity* = *TP* / (*TP* + *FN*) **(3)**

*Specificity* = *TN* / (*TN* + *FP*) **(4)**

*F*1 *score* = 2 × (*Precision* × *Sensitivity*) / (*Precision* + *Sensitivity*) = 2*TP* / (2*TP* + *FP* + *FN*) **(5)**

Statistical comparison of the demographic and clinical data was performed using the Python (Python Software Foundation) SciPy [31] library using the Mann-Whitney U test for continuous variables and the chi-square test for categorical variables. All numerical values are expressed as mean (SD) or n (%).

## Results

### Demographic, Clinical, and Laboratory Information

The patients were classified as belonging to either the event group (n=42) or the event-free group (n=255) (see Table 1). Age, sex, and smoking history were significantly different between the two groups. Fever was the most common initial symptom (249/297, 83.8%), followed by cough (182/297, 61.3%), dyspnea (104/297, 35.0%), myalgia (92/297, 31.0%), and headache (68/297, 22.9%). Compared with the event-free group, the event group exhibited a significantly higher percentage of patients presenting with dyspnea (66.7% vs 29.8%) but a lower percentage presenting with headache (4.8% vs 25.9%). Further, clinical parameters associated with respiratory function and inflammation (eg, oxygen saturation, WBC, and CRP level) were predominantly increased in the event-free group (see Table 1).

**Table 1:** Demographic and clinical data for the event versus event-free data sets.

Characteristic		Total cohort (N=297)	Event-free group (n=255)	Event group (n=42)	*P* value
Sex (female), n (%)		169 (56.9)	155 (60.8)	14 (33)	<.001
Age (years), mean (SD)		60.6 (16.7)	58.7 (16.6)	72.9 (11.9)	<.001
**Smoking status, n (%)**					<.001
	Never smoked	263 (88.6)	233 (91.4)	30 (71)	N/A^a^
	Current smoker	25 (8.4)	14 (5.5)	11 (26)	N/A
	Ex-smoker	9 (3.0)	8 (3.1)	1 (2)	N/A
Diabetes mellitus, n (%)		70 (23.6)	58 (22.7)	12 (29)	.41
Hypertension, n (%)		95 (32.0)	74 (29.0)	21 (50)	.007
Coronary artery calcification, n (%)		39 (13.1)	32 (12.5)	7 (17)	.47
Chronic obstructive pulmonary disease, n (%)		14 (4.7)	8 (3.1)	6 (14)	.002
Chronic kidney disease, n (%)		6 (2.0)	3 (1.2)	3 (7)	.01
Fever, n (%)		249 (83.8)	210 (82.4)	39 (93)	.09
Cough, n (%)		182 (61.3)	157 (61.6)	25 (60)	.80
Dyspnea, n (%)		104 (35.0)	76 (29.8)	28 (67)	<.001
Myalgia, n (%)		92 (31.0)	83 (32.5)	9 (21)	.15
Headache, n (%)		68 (22.9)	66 (25.9)	2 (5)	.003
Systolic blood pressure, mean (SD)		129.9 (19.0)	128.5 (18.1)	138.1 (22.4)	.02
Heart rate, mean (SD)		84.3 (14.4)	83.6 (13.4)	88.3 (18.9)	.10
Respiratory rate, mean (SD)		20.3 (2.9)	20.2 (2.4)	21.3 (4.8)	.28
Oxygen saturation, mean (SD)		96.1 (3.5)	96.5 (2.5)	93.9 (6.9)	.20
White blood cell count (count/µL), mean (SD)		6057.4 (2930.5)	5589.6 (2226.0)	8897.1 (4656.4)	<.001
C-reactive protein (mg/dL), mean (SD)		4.2 (5.8)	3.1 (4.7)	11.0 (7.4)	<.001

^a^N/A: not applicable; the *P* value that was reported for *Smoking status* was based on the chi-square test between the three groups (ie, never smoked, current smoker, and ex-smoker), therefore, it is not reported for each group.

### Analysis of Risk Features

**Figure 3:**
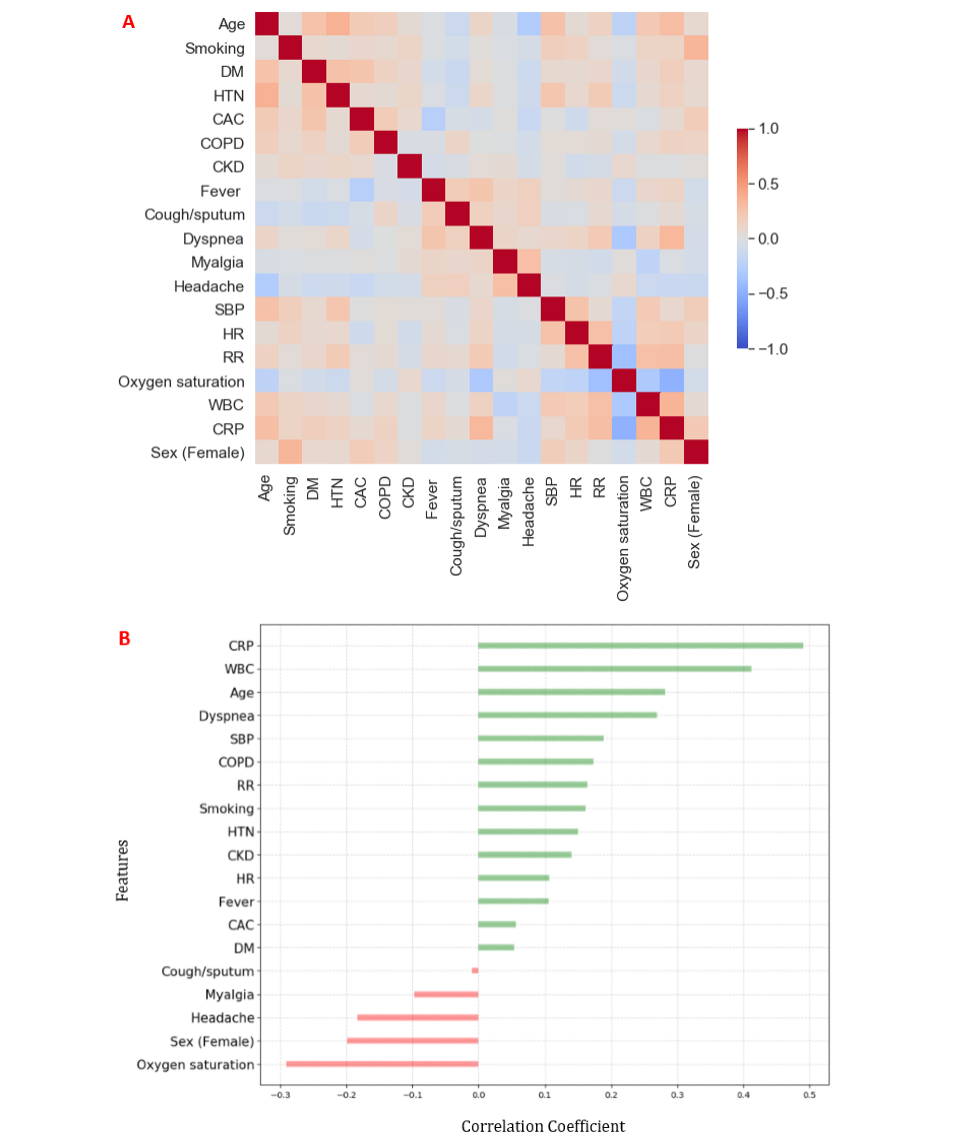
A. Correlation heatmap of clinical features with the endpoint (event vs event-free). B. Diverging bars of important features with endpoint. CAC: coronary artery calcification; CKD: chronic kidney disease; COPD: chronic obstructive pulmonary disease; CRP: C-reactive protein; DM: diabetes mellitus; HR: heart rate: HTN: hypertension; RR: respiratory rate; SBP: systolic blood pressure; WBC: white blood cell count.

We performed correlation tests to determine the clinical features that contributed to the endpoint using a Pearson correlation heatmap [32] (see Figure 3). The heatmap in Figure 3, A highlights potentially important clinical metrics to be considered when constructing a DL model of ANN. The intensive colors of either red or blue indicate a greater correlation magnitude. Figure 3, B also shows the Pearson correlation coefficients between clinical parameters with the endpoint. CRP level and WBC were the most important features having a strong positive correlation with the endpoint. Age was a significant risk factor related to the endpoint, which was in accordance with recent conclusions [33]. Conversely, oxygen saturation and sex (female) were negatively correlated with the endpoint and were identified as significant contributors to the clinical prognosis estimation.

### Performances of Deep Learning Models: ANN Versus CNN Versus ACNN

Performance metrics from the DL models are reported in Table 2. The ANN model only uses clinical metrics without considering CT images (see Multimedia Appendix 1). The ACNN model combined both an ANN with clinical data and a CNN with 3D CT imaging data by concatenation. The reported metrics of the learning models were averaged using 5-fold cross-validation, and a threshold was set to the sigmoid output of 0.5.

Both the ANN and CNN models provided an accuracy greater than 90%; however, their F1 scores were only about 64%, based on the precision and sensitivity. While both models show a potential to differentiate event versus event-free cases, the respective model performances were insufficient to be used in a clinical setting. However, the ACNN model outperformed both models in almost all classification performances. The ACNN model for the NCP lesion achieved the best performance in terms of accuracy (93.9%), sensitivity (80.8%), specificity (96.9%), and AUC score (0.916). These results demonstrate that the combination of clinical information and imaging data can significantly improve the classification performance.

**Figure 4:**
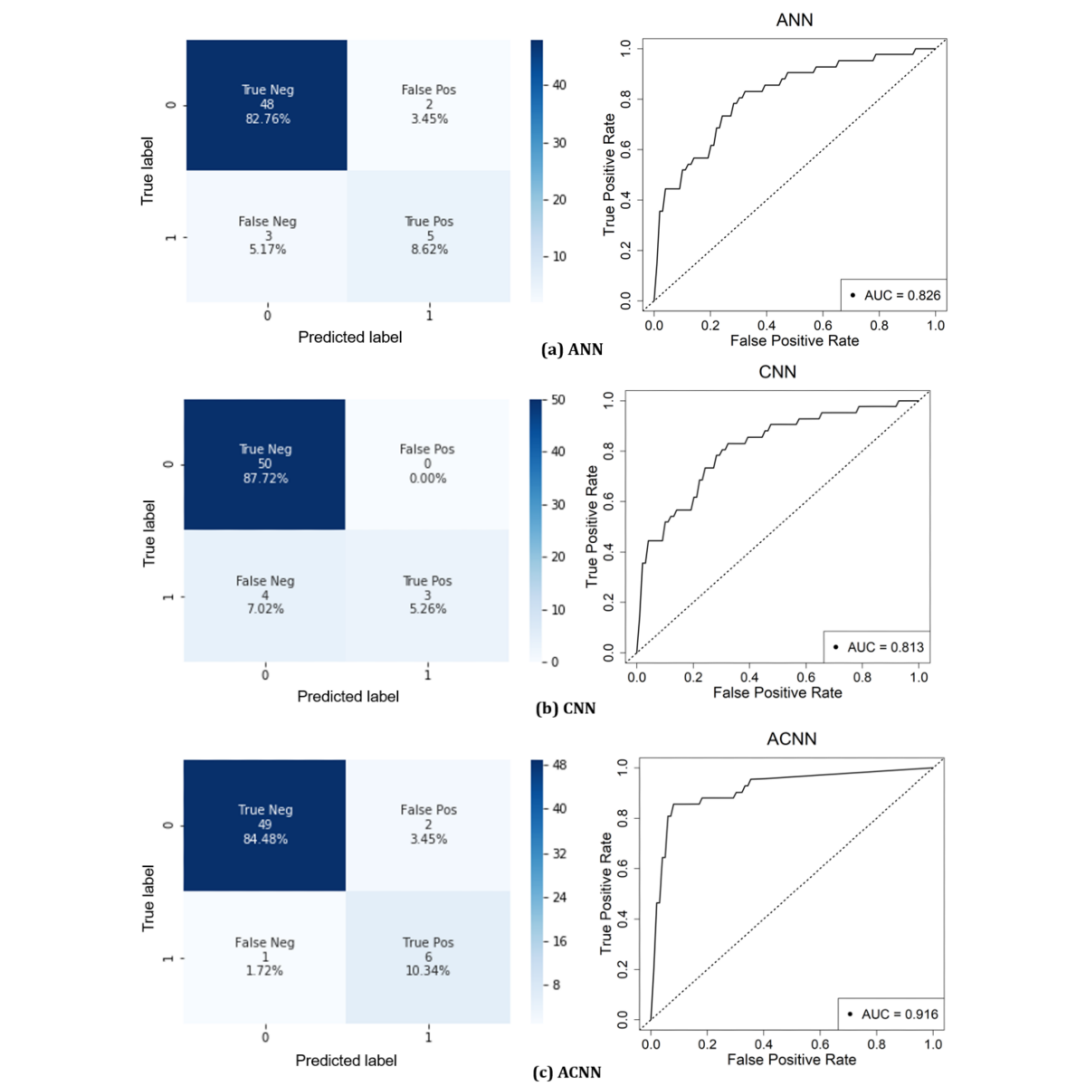
Receiver operating characteristic (ROC) curves and confusion matrices (with a threshold of 0.5) of (a) artificial neural network (ANN), (b) convolutional neural network (CNN), and (c) artificial convolutional neural network (ACNN) models for event and event-free novel coronavirus pneumonia (NCP) lesion data sets. AUC: area under the curve; Neg: negative; Pos: positive.

Figure 4 shows the ROC curves with an AUC score for the ANN, CNN, and ACNN models during testing with the NCP lesion data set. Similar to the prediction accuracy, the AUC score of the ACNN model (0.916) was greater than those of the ANN model (0.851) and the CNN model (0.813). Based on the confusion matrix, the ACNN model produced two FPs (ie, event-free wrongly predicted as event) and one FN (ie, event wrongly predicted as event-free) (sensitivity=80.8%). The FP to FN occurrence ratio for the ANN model was 2:3 and for the CNN model was 0:4. Thus, the ACNN model was much more effective in eliminating FNs. Moreover, compared with the ACNN model, the training times using the ANN and CNN models were shorter (ie, ~3.5 min for ANN vs ~145 min for CNN vs ~150 min for ACNN).

### Effects Caused by the Use of Three Different Imaging Data Sets With ACNN Models

The effects of using different inputs (eg, raw, lung segmentation, and NCP lesion images) were assessed for the ACNN model. The prediction accuracy was 91.6% for raw, 94.3% for lung segmentation, and 93.9% for NCP lesion (see Table 2). The lung segmentation AUC score (0.928) was similar to the NCP lesion AUC score (0.916). However, both performed significantly better than the raw AUC score (0.896). This indicates that lung segmentation and NCP lesions contain more information associated with clinical outcome than other areas.

**Table 2:** Performances of artificial neural network (ANN), convolutional neural network (CNN), and artificial convolutional neural network (ACNN) models for predictions of event versus event-free cases.

Input data and model		Accuracy	Precision	Sensitivity	F1 score	Specificity	AUC^a^ score
**Clinical metrics only**							
	ANN	92.9	85.1	63.9	71.5	94.4	0.851
**NCP^b^ lesion**							
	CNN	91.9	86.7	51.9	64.1	92.6	0.813
	ACNN	93.9	78.3	80.8	78.9	96.9	0.916
**Lung segmentation**							
	CNN	90.6	83.3	45.0	56.0	91.6	0.804
	ACNN	94.3	87.1	74.7	78.1	95.9	0.928
**Raw**							
	CNN	90.3	74.4	50.6	57.3	92.3	0.781
	ACNN	91.6	77.9	63.3	67.1	94.4	0.896

^a^AUC: area under the curve.

^b^NCP: novel coronavirus pneumonia.

### Classification by Other Comparative ACNN Models

Using the NCP lesion image, we constructed three other ACNN models using existing available models (ie, ResNet50, DenseNet121, and InceptionV3). We then compared them with the proposed ACNN model (see Table 3). All models provided similar performances, although the sensitivity was much lower for ResNet50 (78.3%), DenseNet121 (56.1%), and InceptionV3 (59.4%) as compared with the proposed ACNN model (80.8%). The AUC scores of the three online models were also considerably smaller than that of the proposed ACNN model.

Furthermore, we developed a 2D ACNN model using a middle slice. The 2D ACNN model had a final accuracy of 91.9%, a sensitivity of 52.8%, and a specificity of 92.7%. The 2D ACNN model performed worse than the 3D ACNN model, particularly with regard to the sensitivity. The poor performance of the 2D ACNN model is presumed to be related to the loss of the 3D context, proving that discriminative information in all slices improved the prediction performance.

**Table 3:** Performance of the 2D artificial convolutional neural network (ACNN) model and other 3D models, constructed using free source codes available online, for 297 subjects with the novel coronavirus pneumonia lesion data set: prediction of event versus event-free.

Model	Accuracy	Precision	Sensitivity	F1 score	Specificity	AUC^a^ score
ACNN–ResNet50^b^	93.3	75.3	78.3	76.6	96.5	0.900
ACNN–InceptionV3	91.9	78.4	59.4	67.2	93.6	0.814
ACNN–DenseNet121	91.6	88.7	56.1	63.0	93.5	0.826
Our 2D ACNN model	91.9	86.0	52.8	63.8	92.7	0.873
Our ACNN model without cost-sensitivity method	92.3	78.8	69.4	70.1	95.1	0.865

^a^AUC: area under the curve.

^b^ResNet50: residual neural network 50.

### Prognostic of the CNN Model by Grad-CAM Visualization

**Figure 5:**
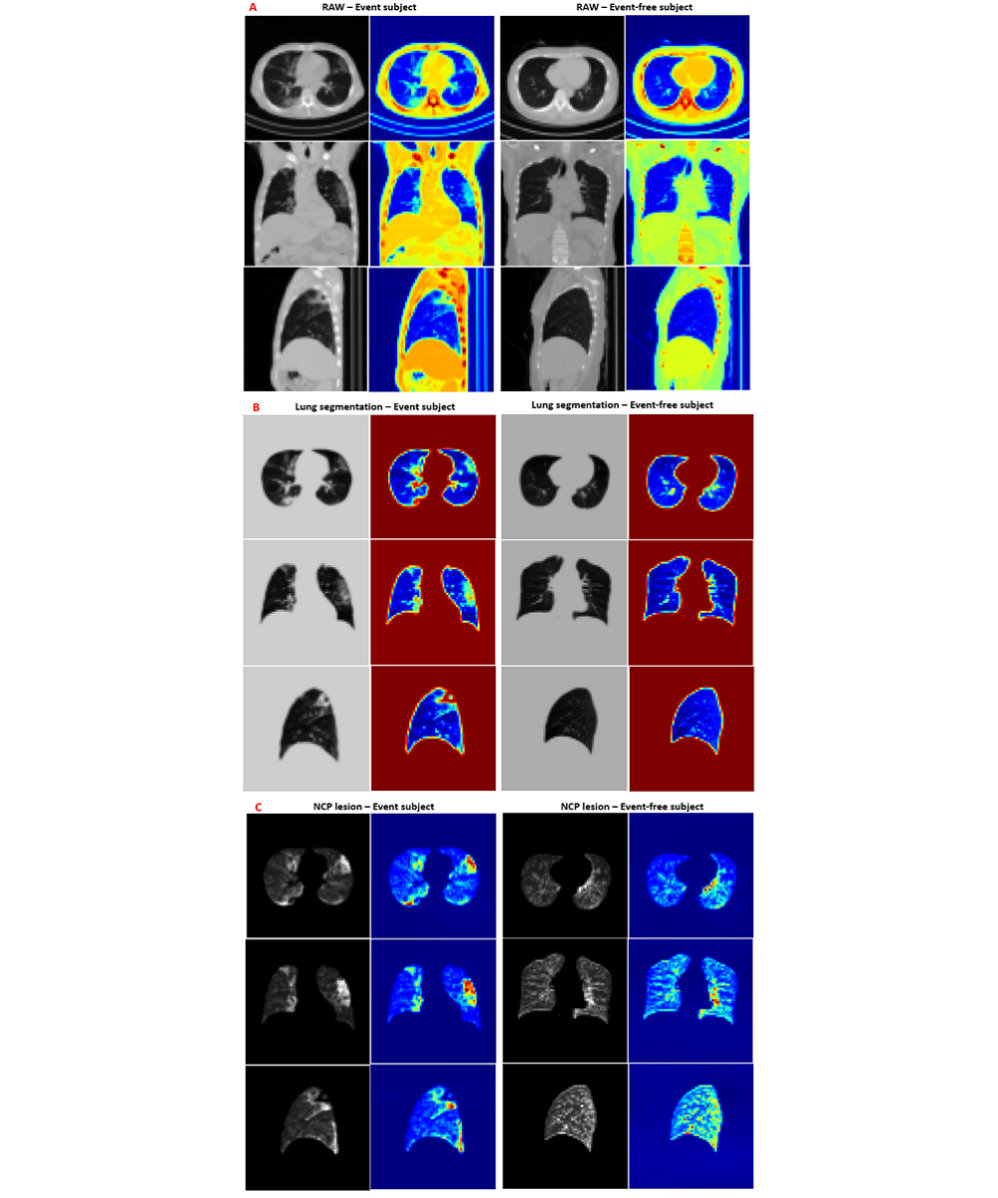
Gradient-weighted class activation mapping (Grad-CAM) heatmap images for representative event and event-free COVID-19 patients with (A) raw computed tomography (CT) images, (B) lung segmentation images, and (C) novel coronavirus pneumonia (NCP) lesion images.

Suspicious lung areas (ie, lesion regions) discovered via the CNN system through the Grad-CAM visualization algorithm made it possible to visualize lung regions that drew the most attention in the CNN model. Figure 5 illustrates the CNN-discovered suspicious lung areas for both event and event-free patients for the three data set types. From Figure 5, A, the Grad-CAM of raw images is able to focus on areas inside the NCP lesions, but almost all of the mapping locates regions outside of the lung at random. Different scanners create different CT domains; therefore, this artificial area may be the source of the FNs and FPs. This effect could be avoided using lung segmentation and NCP lesion images.

Based on Figure 5, B and C, the CNN model discovered sensitive lung regions within the high-attenuation area (ie, brighter regions). The most distinguishing features are the combination of high-level features (ie, GGO, consolidation, and semiconsolidation). From Figure 5, C, we can see that with the combination of lesion features, the obtained activation maps cover the similar highlighted regions as the lung segmentation images. The main difference is that the NCP lesions are more discrete owing to different pixel densities, which contributes to the enhancement of only part of the final features.

We found a high overlap when comparing these CNN-discovered suspicious lung areas with actual abnormal lung areas. This is consistent with radiologist experiences wherein COVID-19 patients have demonstrated lung lesion features. These results suggest that the lesion features have a potential prognostic value for COVID-19 patients and they verify the effectiveness of the NCP lesions.

## Discussion

### Principal Findings

In this study, we developed three DL models for the rapid diagnosis of COVID-19 using clinical data and different CT image types. The identification of high-risk patients is critical because they can progress toward severe or critical illness. Based on our data set, the COVID-19 abnormality manifests itself in various forms and ranges in severity between groups. These abnormalities could be efficiently captured by combining clinical parameters using an ANN model and CT images using a CNN model. Through the mixed ACNN model, we could obtain a high classification accuracy of 94.3% for event versus event-free groups averaged with 5-fold cross-validation. The ACNN model performances using lung segmentation or NCP lesion images achieved accuracies of 94.3% and 93.9%, sensitivities of 74.7% and 80.8%, specificities of 95.9% and 96.9%, and AUC scores of 0.928 and 0.916, respectively. This indicates that lung or NCP lesion images contained high-level features that can effectively represent distinct and abnormal morphological appearances as compared with raw images.

To improve the sensitivity, we applied a cost-sensitive learning method by changing the misclassification cost [29]. This class weighting was achieved using the inverse of the class distribution present in the training data set. Using the cost-sensitivity method, the prediction sensitivity was 80.8%, which was much greater than without using the cost-sensitivity method (69.4%) for the NCP lesion data set. Furthermore, the accuracy and specificity increased by implementing this method (see Table 3).

Zhang et al developed an AI-assisted model using chest CT scans to predict the clinical outcome for COVID-19 patients. They also showed that the clinical outcome exhibited better performance when combined with clinical data: 86.71% sensitivity, 80.00% specificity, and 0.909 AUC score [18]. This is consistent with our result showing that the combination of clinical and imaging information showed better performance. However, our results demonstrated that all ACNN models showed high specificity (94.4%-96.9%) for event prediction in COVID-19 patients (see Table 2). That means that intensive medical treatment is needed if the patient is expected to have a poor prognosis based on an ACNN model. This information may be useful in classifying patients according to risk, particularly in hospitals that are already overloaded owing to the COVID-19 pandemic.

The accuracy for the CNN model using raw images (90.3%) was lower as compared with its accuracy using NCP lesions (91.9%) and lung segmentation (90.6%). This was also true for the ACNN models where the accuracy of the raw images (91.6%) was lower than the accuracy when using NCP lesions (93.9%) or lung segmentation (94.3%). The poor performance of raw images could be attributed to the redundancy around the lungs rather than considering the lung purely, and this extra area can affect the diagnosis. While the recommended chest CT coverage was from the thoracic inlet to the upper abdomen [34], in poor medical environments patients were examined using wide-coverage CT to increase the success of the scan. Therefore, we view a more focused CT image scan and inclusion of the segmentation process as essential when developing a model to predict a clinical outcome.

Existing well-known DL models with deep network structures (ie, ResNet50, DenseNet121, and InceptionV3) were also implemented. It was assumed that these models would be more accurate, as they were developed using lots of imaging data. However, as shown in Table 3, the performances of these models were not as good as those of the proposed ACNN model, which used a relatively simple network. We presume that this is because of the relatively small data set being insufficient to train the complex network of the existing models. Although the first convolutional layer can extract diverse representations through multiple slices, this advantage can be weakened with the increased depth of the model. In other words, deeper networks may not perform better than shallower networks because of the limited data set [35].

The correlations between the COVID-19 outcomes, demographics, clinical parameters, and biomechanical parameters were also evaluated. Identified parameters, such as systolic blood pressure, WBC, CRP level, respiratory rate, heart rate, and oxygen saturation, were viewed as prognostic factors. This is consistent with the prognostic factors seen in severe COVID-19 patients with multiorgan failure. These parameters can assist doctors in quickly screening patients and also ease the significant demand for diagnostic expertise, particularly during a crisis such as a pandemic.

### Limitations and Future Work

Our study had several limitations. First, this was a retrospectively designed study, where the data set size (ie, the number of patients) was small. Moreover, the number of patients that progressed to the severe stage was relatively small (42/297, 14.1%). Therefore, the accuracy and sensitivity of the CNN model were based on CT images that may be affected by the variation in imbalanced data sets. To overcome this imbalance, we first implemented a cost-sensitivity approach. Second, we only included COVID-19 data in this study. However, a real diagnosis model should contain the features to distinguish COVID-19 from other types of pneumonia (eg, flu, viral pneumonia, and bacterial pneumonia). Third, we compared our model with three typical 2D models that were developed into a 3D domain. As these models were designed for 2D images, this comparison did not present the alternatives for the same domain of application. Therefore, the 3D context is important for differentiating between event versus event-free COVID-19 structures, necessitating the development of 3D pretrained models. Fourth, in clinical practice, acute dyspnea is one of the most common symptoms in patients with pulmonary thromboembolism (PTE) resulting in serious consequences. As some of the patients included in this study suffered from acute dyspnea, physicians preferred enhanced CT scans to exclude PTE. In patients with high fever, CT scans with contrast agent were performed to determine fever focus. Although imaging analysis may be affected by the contrast agents used, only 7% of CT scans (21/297, 7.1%) included in this study were performed with a contrast agent. In the pulmonary segmentation technique, large blood vessels and intraperitoneal organs, in which contrast medium is mainly distributed, are removed. Therefore, we believe that the effect of the contrast agents on imaging analysis was minimal. Finally, our data sets were sourced from five hospitals adopting different imaging protocols. The main issues that could be caused by the variety of reconstruction kernels were image noise, artifacts, and changes in the HU values. These variations may affect lung lesion segmentation parts and subsequent calculation results. We controlled for the effect of scanner variation by resampling and normalizing the imaging data. Therefore, these limitations are viewed as potential expansions of this research in future studies. Another potential for future research is to test the generalizability of our models once more patients are enrolled from different centers.

### Conclusions

In summary, our study assessed the imaging and clinical features related to COVID-19 from five centers. Our models suggested that the ACNN model can identify and predict COVID-19 patients at risk of severe status without conducting laboratory tests. We believe our work is meaningful for risk stratification management, which is helpful for alleviating overburdened medical resources while also helping reduce the mortality rate of COVID-19.

## Appendix

Multimedia Appendix 1 Classification performance depending on the number of layers for the artificial neural network (ANN) model.

## Abbreviations

ACNN: artificial convolutional neural network
Adam: adaptive moment estimation
AI: artificial intelligence
ANN: artificial neural network
AUC: area under the curve
CIP: Chest Imaging Platform
CNN: convolutional neural network
CRP: C-reactive protein
CT: computed tomography
DL: deep learning
DRUNET: dilated-residual U-Net
FCN: fully convolutional network
FN: false negative
FP: false positive
GGO: ground-glass opacity
Grad-CAM: gradient-weighted class activation mapping
HU: Hounsfield unit
NCP: novel coronavirus pneumonia
NRF: National Research Foundation of Korea
PTE: pulmonary thromboembolism
ReLU: rectified linear unit
ResNet: residual neural network
ROC: receiver operating characteristic
RT-PCR: reverse transcription–polymerase chain reaction
SegNet: segmentation network
TN: true negative
TP: true positive
WBC: white blood cell count
