# Supplementary material for: Deep Learning Models for Predicting Severe Progression in COVID-19-Infected Patients: Retrospective Study
Source: JMIR Med Inform. 2021 Jan 28;9(1):e24973. doi: 10.2196/24973 (PMC7850779; doi:10.2196/24973)
Supplement: Multimedia Appendix 1 [file medinform_v9i1e24973_app1.docx]

**Table S1. Classification performance depending on the number of layers for the ANN model.**

| Number of layers | Number of nodes | Accuracy | Precision | Sensitivity | F1 | Specificity | AUC |
| --- | --- | --- | --- | --- | --- | --- | --- |
| 1 | 19-1 | 90.2 | 68.9 | 59.4 | 63.3 | 93.5 | 0.794 |
| 2 | 19-10-1 | 91.2 | 75.2 | 56.9 | 64.1 | 93.3 | 0.821 |
| **3** | **19-13-6-1** | **92.9** | **85.1** | **63.9** | **71.5** | **94.4** | **0.851** |
| 4 | 19-14-10-5-1 | 91.9 | 93.1 | 47.8 | 61.1 | 92.1 | 0.800 |
| 5 | 19-15-11-8-4-1 | 91.2 | 76.9 | 54.7 | 63.5 | 92.9 | 0.814 |
| 6 | 19-16-13-10-6-3-1 | 91.2 | 81.0 | 51.7 | 62.1 | 93.0 | 0.724 |
